# Antioxidant Packaging Films Based on Ethylene Vinyl Alcohol Copolymer (EVOH) and Caffeic Acid

**DOI:** 10.3390/molecules25173953

**Published:** 2020-08-29

**Authors:** Francesca Luzi, Luigi Torre, Debora Puglia

**Affiliations:** Civil and Environmental Engineering Department, University of Perugia, UdR INSTM, Strada di Pentima 4, 05100 Terni, Italy; francesca.luzi@unipg.it (F.L.); luigi.torre@unipg.it (L.T.)

**Keywords:** caffeic acid, poly (vinyl alcohol-co-ethylene), antioxidant properties, active packaging

## Abstract

The main objective of this research activity was to design and realize active films with tunable food functional properties. In detail, caffeic acid (CA), a polyphenol with high antioxidant effect, was used as active ingredient in poly (vinyl alcohol-co-ethylene) (EVOH) films at 5 wt.% and 15 wt.% and successfully realized by means of the solvent casting process. Optical, morphological, thermal and mechanical studies were considered to define the effect of the presence of the CA component on the structural properties of the matrix. In addition, moisture content and antioxidant activity were evaluated, to have clear information on the CA effect in terms of functional characteristics of realized food packaging systems. Results from tensile tests showed increased values for strength and deformation at break in EVOH_CA based films. Results from colorimetric and transparency analysis underlined that the presence of caffeic acid in EVOH copolymer induces some alterations, whereas the addition of the active ingredient determined a positive radical scavenging activity of the formulations, confirming the possibility of practically using these polymeric systems in the food packaging sector.

## 1. Introduction

Food packaging comprises a significant part of the packaging industry, so innovations in this sector have been caused and justified by specific applications that require specific properties for the packaging in direct contact with foods. These innovations were motivated mainly by consumer preferences and needs, as well as variations in global trends. The main innovations in packaging, and specifically in the food packaging sector, have been oriented towards the development of ecological systems with active ingredients having antioxidant, antimicrobial and antifungal activities, even being able to reduce, in parallel, environmental pollution and increase shelf-life.

The need to modulate and extend the shelf-life and the freshness of food products is born from the need to increase the storage and transportation time necessary for the containment before sale and consumption [1].

In the last two decades, traditional food packaging was considered unsuitable to guarantee the commercialization and essential shelf-life conditions required by the sector, precisely in the case of fresh or minimally-processed food products [2]. The function of packaging is focused on containing, covering and protection of foods from environmental attacks, such as light radiation, moisture, contaminations and oxidation processes, dangerous for food products susceptible to these external stresses [3]. In order to reduce and obstacle of deterioration and oxidation processes, largely present in delicate/sensitive foods, the presence of inert atmosphere, the use of antioxidant ingredients or the design of a suitable vacuum system packaging technology are the main valid strategies to adopt in the food packaging sector. In this context, the packaging industry is interested in the development of innovative packaging and, thus, the food packaging sector is continuously involved in proposing innovative systems, focusing their attention on the realization of smart and active packaging devices [4].

Active packages are based on interactions with the food’s environment or the food itself. Active food systems offer the possibility of containing active molecules/principles into packages, allowing the reduction of food additives into the food products, maintaining unchanged quality, safety and the organoleptic characteristics of food products and extending the shelf-life of foodstuffs [5,6,7]. This research activity proposed the combination of a poly (vinyl alcohol-co-ethylene) (EVOH) polymer matrix and caffeic acid (CA) active ingredient.

EVOH is a random and semicrystalline copolymer, with high transparency and chemical resistance [8], outstanding interesting barrier properties to gases [9], especially when the content of ethylene in the copolymer is lower than 38 mol.% [9]. EVOH has been largely applied in food-packaging applications, being that this sector is categorized by stringent limitations in terms of gases, aroma, water hydrocarbon permeation and chemical resistance. Barrier and mechanical properties of EVOH systems are largely influenced by the presence of moisture in the copolymer chains, in dry conditions this interesting performance is attributed to high inter- and intra-molecular cohesive energy and semi-crystalline polymer structure [10].

Nevertheless, the main negative aspect of EVOH copolymers is their moisture content sensitivity that determines the reduction of thermal, barrier and mechanical properties [11]. In order to modulate the active properties of polymeric matrices or copolymers, natural extracts and essential oils as active ingredients are used and combined with thermoplastic polymers [12,13,14,15].

Caffeic acid (CA) (3,4-dihydroxycinnamic) is a phenylpropanoid and hydroxy-cinnamate metabolite, present in plant tissues and also in food sources, including blueberries, apples, cider and coffee drink extracts [16]. Caffeic acid is also present in some medications in popular use, based on propolis [17]. It is used as a carcinogenic inhibitor and it possesses antioxidant and antibacterial activity in vitro, contributing to the prevention of cardiovascular and atherosclerosis diseases [18,19]. Natural phenolic antioxidants including caffeic acid have also gained remarkable attention as promising photoprotective agents [20,21] in skin care products, due to their antioxidant activity.

The objective of the present research was the development, production and characterization of EVOH and EVOH_CA thin films for food packaging applications, realized by solvent casting.

In this work, caffeic acid at two different concentrations (5 wt.% and 15 wt.%) was used as active agent. The different amounts of CA were selected on the basis of our previous experience in the use of essential oils and natural and active molecules in polymers [22,23,24,25,26]. The systems were deeply characterized by thermal, optical, morphological and mechanical points of view. Finally, functional properties of relevant importance for packaging sector, such as moisture content and antioxidant behaviour, were determined.

## 2. Results and Discussion

### 2.1. Caffeic Acid Characterizations

**Figure 1:**
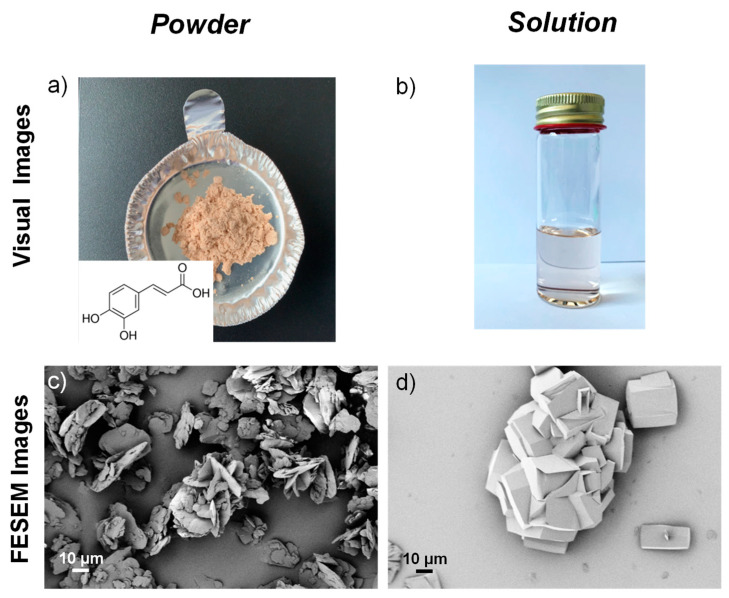
Visual images (chemical structure, see insert of visual image of caffeic acid (CA) powder) of CA powder (**a**) and solution (**b**). FESEM images of caffeic acid in powder (**c**) and after dissolution in 1-propanol/water solution (**d**).

Figure 1a,b show the visual observation (insert chemical structure in a), while Figure 1c,d show FESEM images of caffeic acid in the case of pristine powder and after dissolution in 1-propanol/water. CA is a yellowish powder (Figure 1a), FESEM image (Figure 1c) shows that CA is characterized by an irregular cubic crystal structure, similar to flakes, with smooth surfaces [27]. The flakes of caffeic acid have a length distribution ranging between 5 and 50 μm.

The visual observation and morphological analysis of CA in 1-propanol/water solution are reported in Figure 1b,d, respectively. The CA solution is completely transparent (Figure 1b) maintaining the same colour already observed for the powder, the transparency and solubility are guaranteed by the use of propanol at 70% wt.

The FESEM image of the caffeic acid solution highlights the procedure selected to obtain the dispersion of active ingredients modified the dimension of initial crystals (Figure 1d).

The morphological investigation of CA in solution shows that the active ingredient, after the deposition on the silicon and after the drying procedure, appears with 3D polygonal geometric structures, in most cases agglomerations. The morphological appearance of crystals appeared homogenous and smooth.

**Figure 2:**
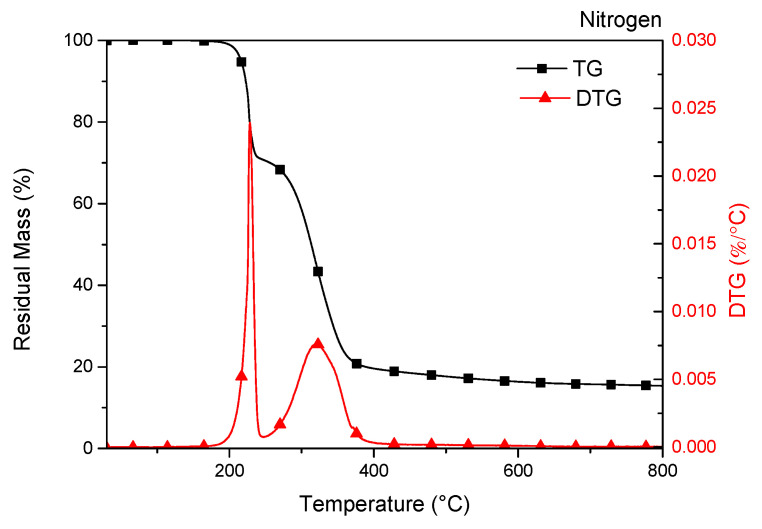
TG (residual mass) and DTG (derivative weight loss) curves of caffeic acid powder under nitrogen atmosphere.

The thermal stability under inert atmosphere of caffeic acid was determined by thermogravimetric analysis (TGA), Figure 2 shows the residual mass (TG) and the DTG curves of caffeic acid. CA shows a multi-step degradation behaviour [27], similar behaviour has been observed in literature for other active ingredients as hydroxytyrosol [28] and gallic acid [22,29].

Profiles of the TG and DTG curves clearly indicate that CA is stable up to 100 °C, while an irregular pattern is visible at higher temperatures, associated with multiple thermal decomposition steps [30]. The caffeic acid starts to decompose (*T_onset_*) at 140 °C [31]. The first stage of weight loss (20.9%), that superimposes melting and degradation of CA [32] was centred at 228.5 °C, while the second decomposition step, characterized by low intensity and centred at 321.4 °C (weight loss 54.9%), can be related to acid decarboxylation [27,32].

### 2.2. Transparency, Colorimetric Analysis and FESEM Investigation of EVOH and EVOH_CA Based Systems

Transparency and colour properties are important characteristics in the food packaging sector. The visual and aesthetic features, in fact, can influence the consumer approval and market success.

**Figure 3:**
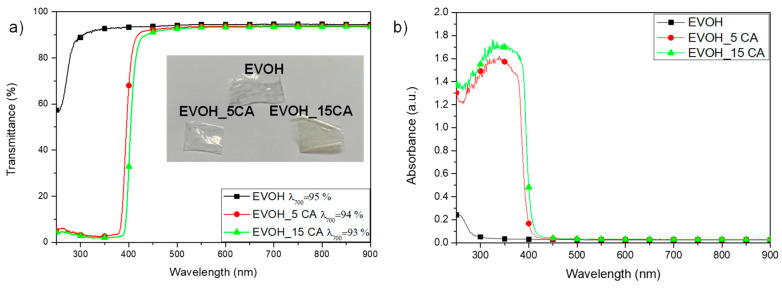
Visual images of the films (insert), transmittance (**a**) and absorbance (**b**) UV-Vis spectra of EVOH based formulations.

Figure 3 shows visual observations (see the insert in the Figure 3) and UV-Vis transmission (a) and absorbance (b) spectra of EVOH and EVOH_CA films. Neat EVOH thin film is a transparent formulation, with a transmittance value of 95% at a visible wavelength of 700 nm. The transmittance trend is maintained stable up to the wavelength value of 350 nm, a slight reduction was registered from 250 to 350 nm [33].

The presence of caffeic acid in EVOH films does not modify the transmittance value detected at 700 nm, the transparency of polymeric films combined with active ingredient (EVOH_5CA and EVOH_15CA) is significantly reduced above 425 nm, this behaviour being related to the addition of CA in the polymeric matrix (see Figure 3b). Furthermore, caffeic acid presents two UV-Vis absorption peaks at 291 and 320 nm [34,35]. The main UV absorbance peak of caffeic acid in EVOH based films is observed at 345 nm (Figure 3b), in accordance with CA absorbance in the UV region, detectable at 365 and 385 nm, as reported in [36].

Colour and gloss values of EVOH and EVOH_CA films are reported in Table 1. The colorimetric and gloss studies have been performed to define the effect of CA on the aesthetic and optical appearance of EVOH films. EVOH is characterized by high lightness value (L* = 98.73 ± 0.16). Moreover, no significant variations of L* parameter have been registered between neat polymeric film and EVOH_CA (Table 1). All of the samples are homogeneous and transparent (as already observed in the UV-Vis analysis, Figure 3), as the registered lightness values (L*) have confirmed, analogous reflection has been done for EVOH combined with catechin and gallic acid [37]. The presence of CA induces a colour variation of a* and b* parameters. A reduction of a* was registered increasing the amount of antioxidant agent, as previously observed in literature using CA in fish gelatine [38]. Positive values have been registered in EVOH film combined with CA for the b* parameter, indicating a slight deviation towards yellowish, desert sand and brown colour hues. The film appearance and colour effect has been induced by the colour of phenolic ingredient, confirming the results of visual observations and UV–Vis analysis (Figure 3, graphs and visual images). The highest ΔE* value for EVOH films has been obtained for EVOH_15CA film (ΔE* = 3.15 ± 0.21).

**Figure 4:**
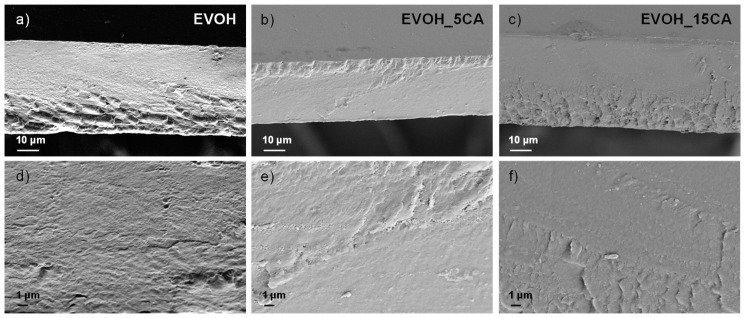
FESEM investigation of fractured surfaces of EVOH based formulations at two different magnifications: EVOH (**a,d**), EVOH_5CA (**b,e**) and EVOH_15CA (**c,f**).

The morphology of EVOH based films was investigated by FESEM. Figure 4 shows the microstructure of fractured cross sections of EVOH based films at two different magnifications. The FESEM analysis was carried out to investigate and evaluate the effect, on microstructure, of the phenolic agent.

EVOH films show an uniform, homogenous and smooth fractured surface (Figure 4a,d) [39], normally obtained for thermoplastic semi-crystalline and well processed polymeric matrices and copolymers [26]. This characteristic highlights the good film-forming processability and properties of EVOH films during the selected process. The presence of CA (5 and 15 wt.%) in cast based films does not induce alterations in terms of uniformity and homogeneity on the fractured surfaces of EVOH_CA films (Figure 4). The homogenous dispersion of CA in EVOH films is also related to the high solubility of CA in the 1-propanol/water solution that has also been demonstrated by the FESEM analysis of the CA ingredient (Figure 1d).

### 2.3. Thermal Characterizations of EVOH and EVOH_CA Based Systems

**Figure 5:**
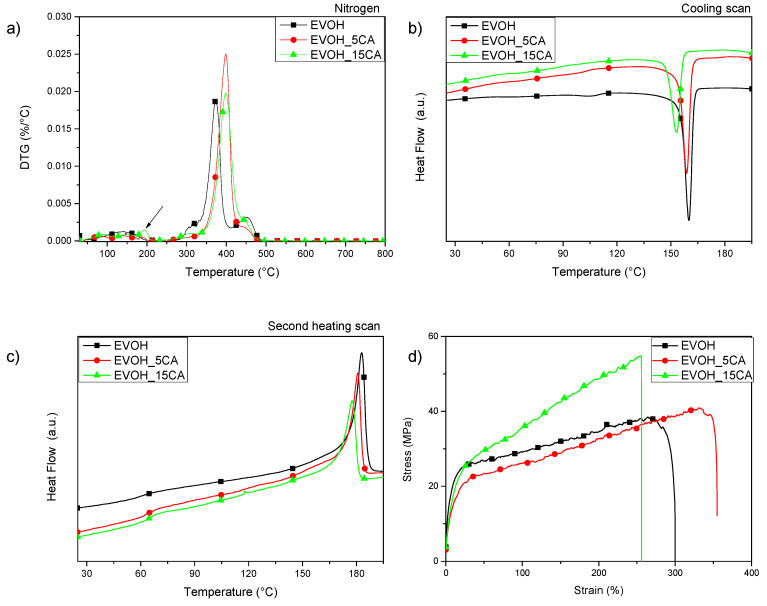
Derivative curves (DTG) (**a**), DSC thermograms cooling (**b**) and second heating scans (**c**) and stress–strain curves (**d**) of EVOH based formulations.

In order to determine the effect of CA presence and content on final thermal characteristics of EVOH realized films, thermogravimetric analysis (TGA) and differential scanning calorimetry (DSC) under inert atmosphere of EVOH based formulations produced by solvent casting have been considered. Figure 5a shows the derivative curves (DTG). The DTG curves of neat EVOH and EVOH_CA films show the presence of a multi-step degradation behaviour (Figure 5a). The first peak centred at low temperature (about 110–120 °C) corresponds to the evaporation of residual solvent (1-propanol/water) [40].

The EVOH film shows two main degradation steps, the first at 373 °C, attributed to the major component fraction, poly (vinyl alcohol) [41,42]. The second one at higher temperature, at approximately 450 °C, could be ascribed to the ethylene component [42]. EVOH_CA based films show a peak centred at 194 °C, the intensity increased with increasing content of CA in the copolymer matrix (see the arrow Figure 5a) and could essentially be associated with the thermal degradation of caffeic acid.

As reported above, CA starts the degradation step (*T_onset_*) at 140 °C [31], while the first and main weight loss of active agent is centred at 228.5 °C [27,32] (Figure 2).

In the presence of CA, the thermal resistance of both formulations is enhanced, a shift of the main degradation temperature (T*_main peak_*) at higher value is registered (T*_main peak_*EVOH = 373 °C, T*_main peak_* EVOH_5CA = 398 °C, T*_main peak_* EVOH_15CA = 399 °C). Observing the thermal profile of the main degradation step of EVOH_CA based films, it is possible to highlight that all the profile peaks are shifted at higher temperatures of about 25 °C. A similar trend has already been detected in previous works where EVOH was combined with gallic acid, umbelliferone and green tea extract [41] and combining other polyphenols in thermoplastic polymeric matrices [26,28]. Thermal properties of EVOH formulations have also been analysed by DSC analysis in order to evaluate the effect of caffeic acid presence and quantity on the crystallization and melting phenomena of the copolymer matrix. The DSC data related to the glass transition, crystallization and melting phenomena determined during the cooling and the second heating scan are summarized in Table 2. The DSC thermograms of the cooling scan and the second heating scan of EVOH based films are reported in Figure 5b and Figure 5c, respectively.

No evident variations have been found in terms of glass transition temperature at cooling and second heating scan adding caffeic acid in EVOH based systems.

Results obtained from the cooling scan highlight the presence of two crystallizations peaks, the first placed at low temperature, approximately at 105 °C for neat EVOH, and the second one centred at a higher temperature (about 160 °C) (Table 2 and Figure 5b); consequently the crystallization degrees are reduced, the lower value of *X_c_* has been found for EVOH_15CA (*X_c_* = (28.1 ± 0.8)%) with respect to the value obtained for EVOH (*X_c_* = (36.6 ± 0.3)%). Furthermore, during the second heating scan, the presence of caffeic acid induced a general decrease of the *T_m_* and the Δ*H_m_* values, this trend is confirmed using different contents of CA [40]. As observed before, the reduction of Δ*H_m_* also determined a decrease of *X_m_.* As reported in the paper by Vannini et al. [43], the addition of low-molecular weight additives in EVOH is a possible strategy to pursue in order to create hydrogen or covalent bonds with the OH groups of the EVOH main chains, thus influencing the crystallization process.

### 2.4. Mechanical Properties of EVOH and EVOH_CA Formulations

The presence and the effect of CA in the EVOH matrix was investigated in terms of mechanical properties. Mechanical data for EVOH and EVOH_CA films were determined from stress–strain curves obtained carrying out tensile analyses at room temperature (RT). The stress–strain curves of different formulations are reported in Figure 5d, while the results are summarized in Table 3.

The presence of CA added at two different concentrations determined modulation and variations of mechanical performance with respect to neat EVOH film (Figure 5d). In detail, the presence of the phenolic ingredient in EVOH film determined an improvement of strength at break (σ_b_). Focusing on the elongation at break and on Young’s modulus values obtained for all EVOH based formulations, it is relevant to observe that the films show an evident ductile behaviour (see Figure 5d and Table 3). In fact, strain at break (ε_b_) increases in EVOH_5CA, while a reduction of ε_b_ was observed in EVOH_15CA with respect to the value registered for neat EVOH. In fact, ε_b_ increases in EVOH_5CA, while a reduction of strain at break was observed in EVOH_15CA. The presence of CA is able to modulate the mechanical performance in the elastic and plastic zones, in relation to the quantity of active ingredient. Adding 5 wt% and 15 wt% of phenolic ingredient a clear reduction of Young’s modulus was observed (E_Young (EVOH)_ = (440 ± 65) MPa, E_Young (EVOH_5CA)_ = (232 ± 11) MPa, E_Young (EVOH_15CA)_ = (207 ± 11) MPa), similar behaviour was observed in literature combining gallic acid and umbelliferone in EVOH based systems [22]. Concluding, EVOH_5CA shows the best mechanical response to tensile stress in terms of elastic and plastic behaviour.

### 2.5. Antiradical Activity and Moisture Content of EVOH_CA Based Systems

**Figure 6:**
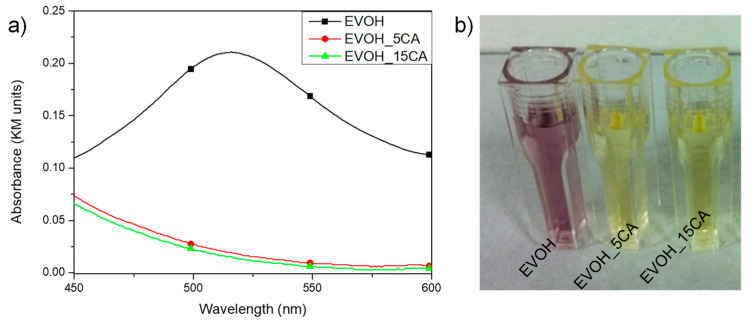
Antioxidant activities of migrating substances for EVOH and EVOH_CA based formulations immersed directly in the methanol solution for 24 h: monitoring of the absorbance band at 517 nm (**a**) and colour variation of the DPPH methanol solution (**b**).

Antiradical activity and moisture content characteristics are considered the main important properties required by polymeric devices applied in the food packaging sector and used in direct contact with food products. The addition of active compounds into polymeric systems determines the antioxidant action. The antioxidant effect of polymeric materials is based on a migration process, into the foodstuff, of active molecules incorporated in polymeric packaging [6]. The continued need for food products with long shelf lives promoted the development of active food packaging systems, this demand was satisfied using active polyphenol molecules in packaging. The antioxidant properties of produced films were determined by the evaluation of the scavenging activity of methanol migration extracts against the DPPH radical. The test represents an indirect method applied to estimate the antioxidant effect of CA on food products. Table 4 and Figure 6a,b summarize the antioxidant effect of migrating substances in methanol for 24 h for polymeric films. Neat EVOH was selected as control. In more detail, Figure 6a shows the monitoring of the absorbance band at 517 nm for migrating substances from EVOH and EVOH_CA based formulations immersed directly in the methanol solution for 24 h, while Figure 6b shows the colour variation of the DPPH methanol solution. CA, and in general the polyphenols, intercept and reduce the effect of the free radical chain of oxidation by donating hydrogen from the phenolic hydroxyl groups. The active process induces the formation of a stable end product, which does not start or extend the lipid oxidation induced in the presence of oxygen and lipids [44].

The incorporation of CA guarantees a clear antiradical scavenging activity (see Figure 6a,b and Table 4), as also confirmed in the literature [17,45]. The higher RSA% value was registered using a low content of active ingredient (RSA (%): EVOH_5 CA = (91.1 ± 0.5)%). Nevertheless, the addition of increasing content of CA (15 wt%) in the EVOH film does not improve significantly the antioxidant response (RSA (%): EVOH_15 CA = (93.6 ± 0.4)%). The phenomenon is due to the presence of CA, characterized by high antioxidant ability [17,45]. Table 5 shows the moisture content (MC) values of EVOH based samples, determined at different times, after 1 and 5 storage weeks at 53% RH and 25 °C. The evaluation of MC and the ability of polymeric films to absorb the H_2_O in the form of moisture represent a crucial focus in food packaging applications, specially using edible films. Pastor and co-authors proposed the development and the characterization of edible films based on hydroxypropylmethylcellulose combined with different concentrations of an ethanolic extract of propolis [46]. In this research, the authors studied how the moisture content influenced the mechanical, water barrier and optical properties of the matrix [46].

The test gives the possibility of estimating the moisture content in different environmental conditions (temperature and relative humidity). The variation/increase of moisture content causes a significant loss in terms of mechanical, thermal and barrier properties at high relative humidity. The absorbed moisture determines an increase of the copolymer free volume in the polymeric chains [10]. The moisture content of the EVOH film was (1.49 ± 0.10)% and (1.53 ± 0.14)% after 1 and 5 weeks, respectively. The equilibrium was achieved in the first week of storage in these specific conditions [22,33,47]. The presence of caffeic acid at two different concentrations in the EVOH film induces a slight increase of absorbed moisture, the equilibrium is guaranteed in the first week of storage. No evident variations of MC% were observed comparing the data obtained from EVOH_5CA and EVOH_15CA at different times. The slight increase of MC% in EVOH_CA based films is also related to the lower crystallinity degrees (*X_c_* and *X_m_*) registered from DSC data (Table 3).

## 3. Materials and Methods

### 3.1. Materials

Poly (vinyl alcohol-co-ethylene) with 32 mol% ethylene content (EVOH 32) (density: 1.19 g/mL at 25 °C, melt index: 3.8 g/10 min (210 °C)), 1-propanol reagent, ≥99.5%, caffeic acid (CA) (C_9_H_8_O_4_, with 98.0% of purity, average, average *M_w_*= 180.16 g mol^−1^) were obtained from Sigma-Aldrich (Milan, Italy).

### 3.2. Characterization of Caffeic Acid

The thermal stability of caffeic acid was determined by using thermogravimetric analysis (TGA, Seiko Exstar 6300, Tokyo, Japan) under nitrogen flow (200 mL min^−1^). Heating scans were performed from 30 to 800 °C at 10 °C min^−1^ and three repetitions of the test have been considered.

The microstructure appearance of CA in powder state and after dissolution in 1-propanol/water solution (10 wt/v.%) was analysed by field emission scanning electron microscope (FESEM, Supra 25-Zeiss). The powder was deposited on conductive adhesive, gold sputtered and analysed, while a few drops of the CA suspension (1-propanol/water (70:30 wt/wt)) were cast on a silicon substrate, dried at room temperature and visualized after gold sputtering. A magnetic stirring at room temperature for 1 h and a sonication bath treatment (Ultrasonic bath-mod.AC−5, EMMEGI, Milano, Italy) for 1 additional hour were applied in order to obtain a uniform dispersion of CA in the 1-propanol based solution.

### 3.3. Production of EVOH and EVOH_CA Systems

EVOH and EVOH_CA based films with 5 and 15 wt.% of caffeic acid (respectively, EVOH_5CA and EVOH_15CA) were prepared by solvent casting method following the procedure reported in literature [48]. EVOH was initially dissolved in 1-propanol/water (70:30 wt/wt.%) in the ratio 8:92 (wt/wt.%) under magnetic stirring at 100 °C for 2 h. EVOH_CA systems were obtained by mixing EVOH solution with a specific amount of the CA previously dispersed in 1-propanol/water. CA dispersion was obtained by applying a two-step procedure: firstly, the CA was dispersed under magnetic stirring in 1-propanol/water solution (10 wt/v.%) for 1 h at room temperature (RT), after that a bath sonication treatment (Ultrasonic bath-mod.AC−5, EMMEGI, Italy) was applied for 1 h at RT. The EVOH solution was cooled down to approximately 60 °C and then mixed with the CA solution by magnetic stirring for 1 h at RT. Finally, EVOH solutions were cast in a Teflon Petri dish and dried in an oven at 60 °C for 3 h. The films (140 mm diameter and 50–70 μm thick) were stored and equilibrated for 2 days in a desiccator by using silica salts, after processing and before characterizations [49].

### 3.4. Characterization of EVOH_CA Based Films

#### 3.4.1. Transparency, Colorimetric Analysis and FESEM Investigation

The transparency of different EVOH films produced by using solvent casting was tested by UV-Vis spectroscopy in the range 250–900 nm by using a Perkin Elmer Lambda 35.

The fractured surfaces of EVOH and EVOH_CA films were analysed by FESEM (Supra 25-Zeiss, Oberkochen, Germany) after gold sputtering and by using an accelerating voltage of 5 kV. The different EVOH formulations were previously freeze-cut in liquid nitrogen, gold-coated with an Agar automatic sputter coater and then characterized.

CIELAB colour space parameters of EVOH produced systems were determined by using a spectrophotometer (CM-2300d Konica Minolta, Tokyo, Japan). The data were examined by using the SCI 10/D65 method, however CIELAB colour, as proposed by the Commission Internationale de 1′Éclairage (CIE 1995), were used. The polymeric films were positioned on a white standard substrate and L*, a* and b* parameters were determined. Samples were examined in triplicate, and three measurements were obtained at random locations on each of the produced films. The total colour difference ΔE* between EVOH based systems was obtained as indicated in Equation (1):

(1) $$\Delta E^{*}=\sqrt{{\left(\Delta L^{*}\right)}^{2}+{\left(\Delta a^{*}\right)}^{2}+{\left(\Delta b^{*}\right)}^{2}}$$

Gloss value was also determined by using the SCI 10/D65.

#### 3.4.2. Thermal Characterizations

Thermal characterization of the EVOH was performed by thermogravimetric measurements (TGA) and by differential scanning calorimetry (DSC). Thermogravimetric measurements were carried out by using a Seiko Exstar 6300. Heating scans from 30 to 600 °C at 10 °C min^–1^ under nitrogen flow (200 mL min^−1^) were performed for each sample.

Differential scanning calorimetric measurements were performed on a TA Instruments DSC Q200 (TA Instruments Inc., New Castle, DE, USA) under nitrogen flow in the range from −25 to 210 °C at 10 °C min^−1^, carrying out two heating and one cooling scans. The glass transition temperature (*T_g_*) was investigated during cooling and second heating scan; crystallization temperature and enthalpy (*T_c_* and Δ*H_c_*) were determined from the cooling scan, whereas the melting temperature and enthalpy (*T_m_* and Δ*H_m_*) were determined from the second heating scan.

The crystallinity degree was evaluated according to Equation (2):

(2) $$\chi =\frac{1}{\left(1-m_{f}\right)}\left[\frac{\Delta H}{\Delta H_{0}}\right]\times 100$$

where ΔH is the enthalpy for melting or crystallization; ΔH_0_ is melting enthalpy for a 100% crystalline EVOH sample and (1 – *m_f_*) is the weight fraction of EVOH in the sample.

The melting enthalpy at 100% of EVOH was calculated according to Equation (3) [50]:

(3) $$\Delta H_{0}=\alpha \Delta H_{0}^{PVA}+\beta \Delta H_{0}^{PE}$$

where ΔH_0_^PVA^ is enthalpy of melting for a 100% crystalline poly(vinyl alcohol) (PVA), taken as 161.1 J g^−1^ [51], while ΔH_0_^PE^ is enthalpy of melting for a 100% crystalline of polyethylene (PE) taken as 290.0 J g^−1^ [50] α and β are the weight fractions of vinyl alcohol (α = 0.68) and ethylene (β = 0.32) in EVOH. ΔH_0_ melting enthalpy for a 100% crystalline EVOH is taken as 202.4 J g^−1^ [22,33].

#### 3.4.3. Mechanical Characterization

Tensile tests of neat EVOH and EVOH_CA systems were carried out to estimate the effect of caffeic acid addition on the mechanical performance of the polymeric matrix. The tests were performed by using a universal test machine (LR30KPlus, Lloyd Instruments Ltd, Bognor Regis, UK) according to UNI ISO 527 rectangular probes about 80 µm thick, a crosshead displacement rate of 5 mm min^−1^ was used. Young’s modulus (E_Young_), the tensile strength (σ_b_) and elongation at break (ε_b_) were determined from the resulting stress–strain curves. The tests were performed at room temperature and at least five samples for each formulation were analysed.

#### 3.4.4. Antiradical Activity

The radical scavenging activity of different films was verified by using a spectroscopic technique according to the method planned in literature [22,52]. The different formulations (0.1 g) were reduced into small dimensions and immersed in 2 mL of methanol for 24 h at RT. An amount of methanol extract (1 mL) was mixed with 1 mL of DPPH in methanol (50 mg L^−1^). The methanolic mixture was maintained at RT in the dark for 60 min. The absorbance was measured at 517 nm using a UV spectrometer (Lambda 35). The DPPH obtained solution of methanol extracted from neat EVOH formulation was used as control. DPPH radical scavenging activity (RSA) was determined according to Equation (4):

(4) $$RSA(\%)=\frac{A_{Control}-A_{Sample}}{A_{Control}}*100$$

where *A_sample_* is the absorbance of sample and *A_control_* is the absorbance of the control.

#### 3.4.5. Moisture Content

The moisture content (MC) of EVOH based formulations was calculated at room temperature and 53% RH. First of all, EVOH and EVOH_CA films were equilibrated in a vacuum oven at 40 °C for 72 h (drying step), after this step the films were placed in desiccators containing Mg(NO_3_)_2_ salts until the constant weight was reached. Three replicates for each formulation for 1 and 5 weeks were evaluated. MC was determined according to the Equation (5):

(5) $$MC(\%)=\frac{W_{Final}-W_{Initial}}{W_{Final}}*100$$

where *W_Final_* is the weight of different materials after 1 or 5 weeks at 53% RH and 25 °C and *W_Initial_* is the initial weight of different formulations after the drying step.

## 4. Conclusions

Poly (vinyl alcohol-co-ethylene) (EVOH) thin systems combined with caffeic acid (CA) at 5 and 15 wt.%, have been realized applying the solvent casting process. Data obtained from optical properties showed transparency of EVOH based films in the visible range stable up to the wavelength value of 350 nm, a slight reduction was registered from 250 to 350 nm only in the presence of caffeic acid (EVOH_CA films). The presence of caffeic acid influenced the thermal behaviour of EVOH based systems; in detail, the thermal degradation stability improved adding CA to the EVOH matrix, while the crystallinity degrees were reduced in active polymeric films. Results from tensile tests showed increased values for strength and deformation at break in EVOH_CA based films. The addition of active ingredient determined a positive radical scavenging activity, confirming the possibility of practically using these polymeric systems in the food packaging sector. EVOH_5CA film represents the best produced formulation. The use of 5 wt.% of CA represents a strategic amount of active ingredient able to guarantee the structural and functional characteristics.

## Figures and Tables

**Table 1:** Colour coordinates of EVOH based systems.

Formulations	L*	a*	a*	∆E*	Gloss (°)
White Control	99.47 ± 0.00	−0.08 ± 0.01	−0.08 ± 0.01	-	121 ± 0
EVOH	98.73 ± 0.16	−0.05 ± 0.00	0.12 ± 0.00	0.77 ± 0.15	158 ± 2
EVOH_5CA	98.89 ± 0.10	−0.25 ± 0.01	0.43 ± 0.03	0.80 ± 0.06	218 ± 3
EVOH_15CA	98.45 ± 0.28	−1.06 ± 0.03	2.73 ± 0.16	3.15 ± 0.21	188 ± 5

**Table 2:** Mechanical properties of EVOH based systems.

**Formulations**	**Cooling Scan**							
	***T_g_* (°C)**	***ΔH’ _c_* (J g^−1^)**		***T’_c_* (°C)**	***ΔH”_c_* (J g^−1^)**	***T”_c_* (°C)**		***X_c_* (%)**
EVOH	61.0 ± 0.7	2.2 ± 0.2		104.9 ± 0.4	71.8 ± 0.4	159.9 ± 0.2		36.6 ± 0.3
EVOH_5CA	60.2 ± 1.0	0.9 ± 0.2		98.5 ± 1.1	59.3 ± 3.3	158.2 ± 0.8		31.3 ± 1.8
EVOH_15CA	60.2 ± 0.4	-		-	48.3 ± 1.4	153.6 ± 0.6		28.1 ± 0.8
**Formulations**	**Second Heating Scan**							
	***T_g_* (°C)**		***ΔH_m_* (J g^−1^)**		***T_m_* (°C)**		***X_m_* (%)**	
EVOH	64.6 ± 0.3		83.2 ± 0.1		182.9 ± 0.1		41.1 ± 0.1	
EVOH_5CA	66.0 ± 2.3		68.3 ± 0.3		178.7 ± 2.7		35.3 ± 0.1	
EVOH_15CA	66.2 ± 2.1		55.7 ± 0.8		173.7 ± 1.5		32.4 ± 0.5	

**Table 3:** Mechanical properties of EVOH based films.

Formulations	σ_b_ (MPa)	ε_b_ (%)	E_Young_ (MPa)
EVOH	45 ± 4	265 ± 28	440 ± 65
EVOH_5CA	41 ± 3	310 ± 26	232 ± 11
EVOH_15CA	58 ± 4	263 ± 11	207 ± 11

**Table 4:** Radical scavenging activity (RSA) of EVOH based systems.

Formulations	Radical Scavenging Activity, RSA (%)
EVOH	0
EVOH_5CA	91.1 ± 0.5
EVOH_15CA	93.6 ± 0.4

**Table 5:** Moisture content (MC) of EVOH based systems.

Formulations	MC (%) @ 1 Week	MC (%) @ 5 Week
EVOH	1.49 ± 0.10	1.53 ± 0.14
EVOH_5CA	1.97 ± 0.10	1.98 ± 0.05
EVOH_15CA	1.98 ± 0.09	2.01 ± 0.08
